# Relevance and Clinical Significance of Magnetic Resonance Imaging of Neurological Manifestations in COVID-19: A Systematic Review of Case Reports and Case Series

**DOI:** 10.3390/brainsci10121017

**Published:** 2020-12-21

**Authors:** Anisa Chowdhary, Roshan Subedi, Medha Tandon, Sijin Wen, Jenil Patel, Saurabh Kataria, Sarah Peterson, Ronald Gwinn, Mahmoud Elkhooly, Apoorv Prasad, Lalit Nirwan, Amelia Adcock, Shitiz Sriwastava

**Affiliations:** 1 Institute of Nuclear Medicine and Allied Sciences, New Delhi 110054, India; anisaanila@gmail.com; 2 Institute of Medicine, Kathmandu P.O.BOX 1524, Nepal; roshansubedi123@gmail.com; 3 Safdarjung Hospital, New Delhi 110029, India; medhatandon22@gmail.com; 4 Department of Biostatistics, West Virginia University, Morgantown, WV 26506, USA; siwen@hsc.wvu.edu; 5 Department of Epidemiology, Fay W. Boozman, College of Public Health, University of Arkansas for Medical Sciences, Little Rock, AR 72205, USA; drjenilpatel@gmail.com; 6 Department of Neurology, University of Missouri Healthcare, Columbia, MO 65212, USA; saurabh.kataria27@gmail.com; 7 School of Medicine, West Virginia University, Morgantown, WV 26506, USA; sipeterson@mix.wvu.edu (S.P.); rlbealeii@mix.wvu.edu (R.G.); 8 Department of Neuropsychiatry, Minia University, Minia 61519, Egypt; elkhoolymahmoud27@outlook.com; 9 Department of Neurology, Berkeley Medical Center, West Virginia University, Morgantown, WV 26506, USA; apoorv.prasad@wvumedicine.org; 10 Meditrina Institute of Medical Sciences, Nagpur, Maharashtra 440012, India; lalitnirwan@gmail.com; 11 Department of Neurology, Rockefeller Neuroscience Institute, West Virginia University, Morgantown, WV 26506, USA; akadcock@hsc.wvu.edu; 12 Department of Neurology, Wayne State University, Detroit, MI 48201, USA

**Keywords:** SARS CoV-2, COVID-19, stroke, neuroimaging in COVID-19, MRI in COVID-19

## Abstract

We performed a systematic literature review of neuroimaging, predominantly focusing on magnetic resonance imaging (MRI) findings associated with neurological manifestations of coronavirus disease-2019 (COVID-19). We screened articles from PubMed, Google Scholar and Scopus, looking for reports that would potentially have neuroimaging findings in patients with COVID-19. Data analysis was performed with patient-based data based on the availability of clinical characteristics and outcomes for each individual patient from the studies. Chi square and Wilcoxon rank-sum tests were used to report COVID-19 severity and outcomes based on neurological imaging indicators and pathophysiology. A total of 171 patients with COVID-19 having neurological complications, from 134 studies, were identified in our review. The most common neuroimaging finding was ischemic stroke (62, 36.2%) cases, followed by CNS inflammatory disorder (44, 25.7%), and hemorrhagic stroke (41, 24.0%). Around 51% of all the fatal COVID-19 cases had an ischemic stroke. Among patients with ischemic stroke, the mean age of those who suffered from COVID-19 infection was 57.5 years (SD = 15.4) whereas it was 50.7 years (SD = 15.1) among those without stroke/other diagnosis. Fatality was more common in patients with ischemic stroke compared to those with other diagnosis (40% vs. 22%, *p* = 0.011). The most frequently published neuroimaging findings in patients with COVID-19 were ischemic stroke, CNS inflammatory disorder, and hemorrhagic disorder. In those studies, ischemic stroke was associated with fatality, and was more frequently seen in older patients. Based on our findings, early usage of MRI in COVID-19 patients may be recommended.

## 1. Introduction

The Coronavirus disease 2019 (COVID-19) outbreak began in Wuhan, China, in December 2019, and has rapidly spread around the world to be declared as a pandemic. As of 31 October 2020, over 45 million COVID-19 cases with more than one million deaths have been reported globally [1,2].

Several new studies have shown neurological complications to be associated among patients with COVID-19 [3,4,5,6]. A study of 214 hospitalized patients in Wuhan reported that 36.4% of patients had neurological symptoms, including dizziness, headache, impaired consciousness, and acute cerebrovascular events [6]. A systematic review of studies reported that one-third of COVID-19 patients were found to have neurological manifestations [7]. Some patients with neurological symptoms have normal imaging findings, while others have findings like stroke (both ischemic and hemorrhagic), cerebral venous thrombosis, encephalitis, microbleed, vasculitis, demyelinating disorders such as acute disseminated encephalomyelitis (ADEM), and encephalopathy, on computed tomography (CT) and magnetic resonance imaging (MRI) [4,5,8,9,10]. Currently, the understanding of the mechanism of these symptoms in COVID-19 patients is sparse, and it is not clear if these symptoms are due to direct viral invasion or indirect neuroinflammatory response. Intense systemic inflammatory response can lead to eventual disruption of the blood–brain barrier (BBB) causing increased permeability to inflammatory cytokines and leading to a cascade of immune cells within the central nervous system (CNS). This is due to the underlying critical illness and systemic complications [11,12,13].

Although COVID-19 typically affects the lungs, clinicians and radiologists should be mindful of the possible concomitant neurological presentations and neuroimaging findings in the affected patients. Definite neuroimaging protocols are not yet established and there is an evident need for a thorough documentation of the neuroimaging findings associated with COVID-19. MRI is the best non-invasive imaging modality to visualize the nervous system and aid in diagnosis of neurological complications in COVID-19 [5,8]. In this study, we conducted a systematic review to collect, analyze, and summarize the neurological findings in the MRI among patients with COVID-19 from studies published worldwide. This review also serves the purpose of being one of the very few literatures on the relevance and clinical importance of the neuroimaging findings in patients with COVID-19, which additionally could also be added to the database for the research to carry out further studies.

## 2. Material and Methods

### 2.1. Study Selection and Criteria

Using the keywords “COVID-19 and CNS”, “COVID-19 and PNS”, “SARS-CoV-2 and CNS”, “COVID-19 and neurological manifestation”, “SARS2 and neurological manifestation”, “COVID-19 and Neuroimaging”, we searched databases including PubMed, Google Scholar, and Scopus from 1 December 2019 to 30 September 2020. Two reviewers independently performed the literature search. Neuroimaging findings and pathophysiology comparisons that were considered within the scope of our review were mainly focused on, but not limited to, ischemic disorder, hemorrhagic disorder, disorders of the CNS and peripheral nervous system (PNS), encephalitis, encephalopathy, olfactory bulb and cranial nerve enhancement, and Guillian-Barre Syndrome (GBS) variants, and their association with severity and outcomes for COVID-19. Neurological manifestations, which were considered minor and thus excluded from this analysis, were as follows: no specific neurologic diagnosis, nerve pain, dizziness, and skeletal muscle injury. Furthermore, we checked the references of the included studies in order not to miss any other eligible studies. Severity of COVID-19 was measured using Infectious Disease Society of America/American Thoracic Society (IDSA/ATS) criteria [14]. We used the preferred reporting items for systematic reviews and meta-analyses (PRISMA) for our study [15].

Our final inclusion and exclusion criteria in order to incorporate the studies were:

Inclusion criteria
  The inclusion criteria for the published studies were: (1) Patient age ≥ 18 years; (2) COVID-19 diagnosis confirmed by real-time reverse transcription polymerase chain reaction (RT-PCR) nasopharyngeal or serum antibody IgG test; (3) Established neurological diagnosis in the patients with COVID-19; (4) Neuroimaging findings of CNS and PNS complications not accounted for by another neurological process.
Exclusion criteria
  The exclusion criteria for the studies were: (1) Duplicate studies which involved repetition of cases; (2) Studies in languages other than English; (3) Studies with no individual data on severity and/or fatality of COVID-19, and (4) Studies with missing clinical information.

Cases were categorized into the following groups: (i) Ischemic disorders including ischemic stroke: both large vessel and small vessel, cardio-embolic, stroke of unknown origin and dural sinus venous thrombosis; (ii) Hemorrhagic disorders including subarachnoid hemorrhage, large and small intracranial hemorrhage, and microhemorrhages; (iii) GBS and its variants; (iv) Encephalitis and meningoencephalitis; (v) Encephalopathy; (vi) CNS inflammatory disorder including multiple sclerosis (MS) exacerbation, acute disseminated encephalomyelitis (ADEM), vasculitis, cytotoxic lesion of corpus callosum (CLOCC) or Mild encephalitis/encephalopathy with reversible splenial lesion (MERS), posterior reversible encephalopathy syndrome (PRES), optic neuritis (ON), transverse myelitis, acute hemorrhagic necrotizing encephalopathy (AHNE); (vii) Olfactory bulb enhancement; (viii) Other cranial nerve enhancement; and (ix) others which includes parenchymal leptomeningeal enhancement and nonspecific findings such as fluid-attenuated inversion recovery (FLAIR) hyperintensity in deep white matter. The cases were further classified based on underlying neurological pathophysiology into: Vascular pathology (including ischemic disorders and hemorrhagic disorders) and CNS inflammatory pathology (including MS exacerbation, ADEM, ON, CLOCC or MERS, transverse myelitis, and AHNE). While we reviewed findings of CT scan, positron emission tomography (PET) scan and MRI scan, we only considered MRI scan findings in our analysis as it was the most commonly reported and it provided the best method of characterizing neuro-imaging findings in COVID-19 infections.

### 2.2. Quality Assessment

The critical appraisal checklist for case reports provided by the Joanna Briggs Institute (JBI) was used to perform assessment of overall quality of case series and case reports [16].

### 2.3. Data Acquisition

From the selected studies, we extracted the following data for our analysis: study type, date of publication, age, gender, clinical presentation of COVID-19, diagnostic tests for SARS-CoV-2 infection including RT-PCR nasopharyngeal and serum antibodies, and imaging markers including MRI scan and severity of COVID-19 (based on IDSA/ATS criteria) [14].

### 2.4. Data Analysis

We conducted statistical analysis to report demographic characteristics such as age, gender, severity and outcomes of all COVID-19 cases presenting with neurological manifestations. Pooled descriptive analyses were performed to assess differences in these markers among groups including severe vs. non-severe, fatal vs. non-fatal, vascular vs. CNS inflammatory disorders. Data analysis was performed with patient-based data based on the availability of characteristics and outcomes for each individual patient from the studies. In particular, we conducted the following analyses: (1) Severity and outcomes of COVID-19 based on neuroimaging findings confirmed through MRI; (2) Age distributions of all COVID-19 patients based on neurological diagnoses through MRI findings; and (3) Severity and outcome status of COVID-19 by type of neurological pathophysiology. Chi square test and Wilcoxon rank-sum test were used in the data analysis for categorical and continuous variables, respectively. All statistical tests were two-sided and a *p*-value < 0.05 implied the statistical significance in this study. Statistical analysis was performed using SAS (version 9.2) and R software (version 3.6.3, R foundation, Vienna, Austria).

## 3. Results

**Figure 1:**
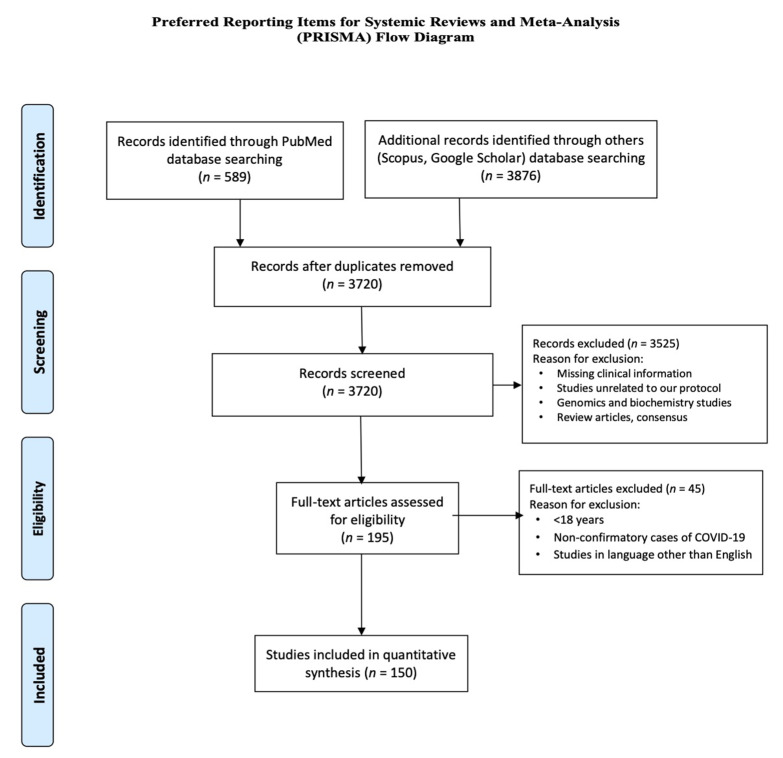
PRISMA flow diagram of systematic review. The flow diagram depicts the flow of information through the different phases of the systematic review. It maps out the number of records identified, included and excluded, and the reasons for exclusions.

Based on our search criteria, we found a total of 4465 studies from all the databases including PubMed (*n* = 589), Google Scholar (*n* = 3720) and Scopus (*n* = 156); 745 of these were identified as duplicates. Finally, we screened 934 studies for title and abstracts, and reviewed 195 full-text studies in accordance with our study objective. Finally, we conducted a systematic review and quantitative analysis of 134 studies (comprising of 171 patients) in accordance with our inclusion and exclusion criteria. Of the 134 included studies, 106 were case reports, 28 were case series. Additionally, 16 retrospective observational studies were not included in statistical analysis but were reported separately, as the substantial data for severity and fatality of COVID-19 was not available from these studies (Figure 1)

Table 1 displays the demographic characteristics of 171 patients with COVID-19 from the 134 studies identified in our review. The most common neuroimaging finding observed in our study was ischemic stroke (*n* = 62, 36.2%), followed by CNS inflammatory disorder (*n* = 44, 25.7%), hemorrhagic stroke (*n* = 41, 24%), encephalitis (*n* = 24, 14%), encephalopathy (*n* = 11, 6.4%), and GBS (*n* = 3, 2%). Over half of the cases (*n* = 96, 56.2%) were categorized to have severe COVID-19, while the remaining (*n* = 75, 43.8%) were categorized to have non-severe COVID-19. An outcome of around 30% of the cases (*n* = 49) were reported as fatal.

Table 2 shows the severity and outcomes of COVID-19 based on neurological pathophysiology confirmed through MRI findings for patients included in statistical analysis. Overall, half of the fatal COVID-19 cases (*n* = 25) reported having an ischemic stroke, based on their MRI findings (*p* = 0.011). We found that fatality was more common in patients with ischemic stroke with 40% (25/62) compared to 22% (24/109) among those with non-ischemic stroke/other diagnosis (*p* = 0.011). Furthermore, among patients with hemorrhagic stroke, the majority of the patients (30/41) had a severe infection, while only 51% (66/130) of the patients without a hemorrhagic stroke developed a severe COVID-19 disease. Additionally, we observed that a greater proportion of patients with hemorrhagic stroke reported a fatal COVID-19 condition (*n* = 17, 41%) compared to those with a non-hemorrhagic stroke (*n* = 32, 25%) (*p* = 0.015). None of the 96 patients with a severe COVID-19 infection had evidence of olfactory bulb enhancement on MRI. However, three patients (4%) with non-severe COVID-19 indicated olfactory bulb enhancement on MRI (*p* = 0.048). Similarly, cranial nerve enhancement was not observed in any patients with a severe COVID infection but was seen in 8% of the non-severe cases (*p* = 0.005).

Table 3 displays the age distributions of all COVID-19 patients based on neurological diagnoses through MRI findings. We tabulated the mean age by outcome and severity of COVID-19, along with distributions by each neurological diagnosis. We found significant differences with respect to mean age among patients with ischemic stroke and olfactory bulb enhancement. Among patients with ischemic stroke, the mean age of COVID-19 patients was 57.5 years (SD = 15.4), significantly different compared to 50.7 years (SD = 15.1) among those without stroke/other diagnosis (*p* = 0.005). Among patients showing olfactory bulb enhancement, the mean age was 29 years (SD = 5.3), significantly lower than 53.6 years (SD = 15.3), among those without olfactory bulb enhancement.

Table 4 displays the sub-analysis of severity and outcome status of COVID-19 by type of neurological pathophysiology. Of the 90 patients with a vascular pathology (including ischemic, hemorrhagic stroke, dual sinus venous thrombosis, hypoxic ischemic encephalopathy (HIE), microhemorrhages), 38% (*n* = 34) reported a fatal outcome as compared to 19% (*n* = 15) who did not report vascular pathology (*n* = 81) (inclusive of ADEM, CNS vasculitis, CLOCC, PRES, MS exacerbation, ON, AHNE), *p* = 0.005).

We explored further literature to report grouped patients that were not included in our study analysis. Table 5 displays the MRI findings and associated neurological manifestations along with severity and outcomes for COVID-19 for all the observational studies included in our review. In sixteen observational studies, 681 patients had findings on neuroimaging [3,5,8,9,10,17,18,19,20,21,22,23,24,25,26,27]. Of those, the most common neuroimaging findings were: ischemic stroke/infarction including lacunar infarct (201, 29.5%) followed by hemorrhagic stroke/ICH (77, 11.3%), encephalitis and encephalopathy (40, 5.9%), and microhemorrhages (36, 5.2%), which is similar to our findings. CNS features (667, 97.9%) were more common than PNS features (14, 2.1%). Severity and fatality data were unavailable for most of these studies, and thus clinical significance could not be analyzed.

## 4. Discussion

In one of most comprehensive reviews on neurological imaging findings and COVID-19 according to our knowledge, we found that more than one third of hospitalized patients with COVID-19 developed some form of neurologic symptoms. These included headache, dizziness, myalgia, alteration of consciousness, anosmia and dysgeusia, strokes, and seizures [4,6,7]. Severe infections were associated with greater neurologic involvement [6,28]. In this paper, we reviewed the CNS and PNS radiological findings in patients with COVID-19 and described the neuroimaging findings in COVID-19 patients and assessed its clinical significance.

Ischemic stroke (36%) was the most common neuroimaging finding in this study. Intense inflammatory changes and cytokine storm triggers hypercoagulability in COVID-19 patients, which, along with vascular endothelial injury, is thought to promote stroke in these patients [29]. Although more patients with severe COVID-19 reported having an ischemic stroke in our study, there was no significant association between ischemic stroke and the severity of COVID-19. The possible explanation could be that ischemic stroke might result from viral pathogenesis and not merely an occurrence in critically ill patients. The fatality in COVID-19 patients was significantly higher in patients with ischemic stroke (40%) compared to patients without ischemic stroke. A systematic review by Tan et al. reported a mortality rate of 38% in their study [30], which is similar to our research.

CNS inflammation (25.7%), hemorrhagic stroke (24%) and encephalitis (14%) were other common neuroimaging findings observed in our study. Direct invasion of the brain by the SARS-CoV-2 virus or immunological response to the virus is implicated in the pathogenesis of encephalitis [31]. Anticoagulation and hemorrhagic transformation of ischemic stroke are thought to cause hemorrhagic stroke [32]. There was a significant association between the presence of hemorrhages on imaging and the severity of the COVID-19, with 73% of hemorrhages being associated with severe disease. This could be because patients with severe disease may develop severe neurovascular injury and are likely to receive multiple interventions like anticoagulation, leading to hemorrhages. A review by Pan et al. also reported that the prevalence of a hemorrhage was more specific for severe COVID-19 patients [33].

**Figure 2:**
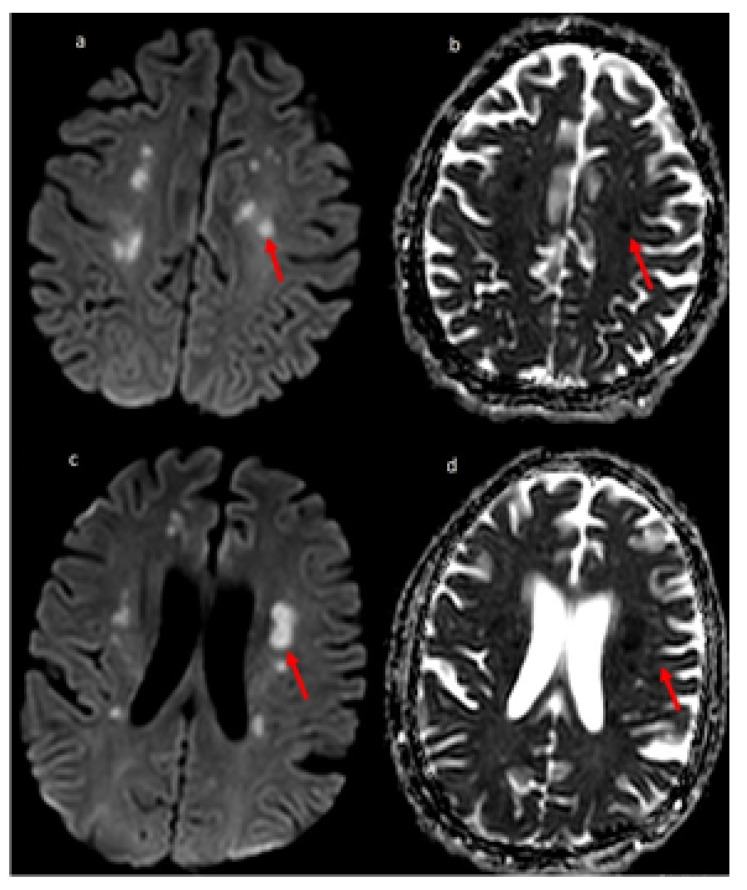
51-year-old man COVID-19 RT-PCR positive presented with dysarthria and change in mentation. MR Diffusion image (**a**) showing multiple foci of restricted diffusion (red arrow) involving centrum semiovale with the corresponding hypointense signal on ADC image (**b**), suggestive of embolic infarcts. (**c**) Showing multiple small foci of restricted diffusion (red arrow) involving centrum semiovale and periventricular white matter with the corresponding hypointense signal on ADC image (**d**), suggestive of embolic infarcts.

**Figure 3:**
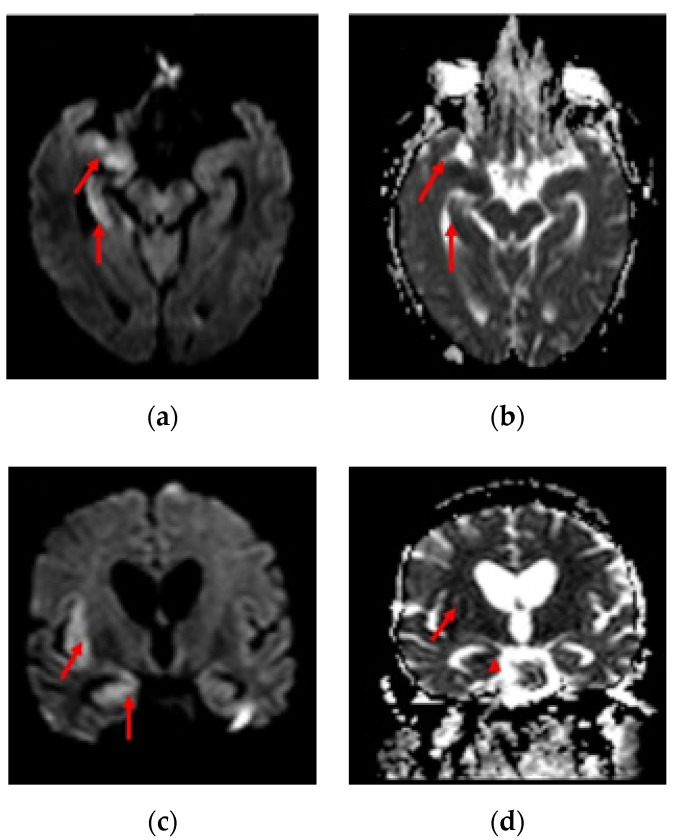
54-year-old man with COVID-19 RT-PCR positive and history of fever, cough for 7 days with recent onset headache and vomiting. MRI DWI (**a**) and ADC (**b**) showed diffusion restriction with corresponding hypointensity on ADC (red arrow) in the medial aspect of right anterior temporal lobe, hippocampus and sylvian cortex. MRI DWI (**c**) and ADC (**d**) showed diffusion restriction with corresponding hypointensity on ADC (red arrow) in the medial aspect of right anterior temporal lobe, hippocampus and sylvian cortex.

Overall, vascular pathology seen in imaging was significantly associated with fatality. A retrospective study by Benussi et al. also concluded that COVID-19 in patients with the cerebrovascular disease had significantly higher mortality than in-patients without COVID-19 [34]. The inflammatory pathology was significantly associated with the severity of the disease (refer Table 4 and Table 5). Severe COVID-19 patients may have developed neurological inflammation due to an intense inflammatory response or immunological phenomenon [13,35,36]. We present the MRI sections of two COVID-19 patients, one with multifocal ischemic stroke and another with encephalitis in Figure 2 and Figure 3, respectively.

Anosmia is a common neurological manifestation of COVID-19 patients and is thought to be mediated by microvascular pathology due to the SARS-CoV-2 virus [37,38]. In our study, the imaging finding of olfactory bulb enhancement was seen in a very small fraction of patients (2%). This may be because most patients with mild–moderate COVID-19 do not routinely undergo neuroimaging evaluation, leading to an underreporting of these findings. Additionally, patients with smell disorders due to nasal obstruction or rhinorrhea may have normal findings in imaging. Olfactory bulb enhancement was seen in only three patients, all of which were non-severe COVID-19 patients. Similarly, six patients with non-severe infection had enhancement of other cranial nerves (refer Table 4 and Table 5). None of the patients with severe COVID-19 demonstrated olfactory nerve enhancement or cranial nerve enhancement. This is similar to the reports by the study by Pan et al., where cranial nerve abnormalities were exclusively seen in patients with mild infection [33].

Older patients (mean age 57.5 years) were significantly more likely to show ischemic stroke than younger patients. This is an important finding as it might suggest an underlying vascular pathology in the older population. Similarly, a review by Tan et al. reported the mean age of 63.4 years for COVID-29 patients with ischemic stroke [30]. Most strokes in non-COVID-19 patients usually occur in people with age >65 years [39], which is much higher than what we found in our study. Anosmia or ageusia was significantly more prevalent in younger patients (mean age 36.5 years) in a study by Lee et al. [40] Our study also showed that the younger patients were more likely to have olfactory bulb enhancement (mean age 29 years) compared to older patients (refer Table 3).

Our study had several limitations. First, a subset of neuroimaging findings, particularly those in the critically ill elderly patients, may be due to comorbidities or other factors and may not be directly related to the COVID-19 infection. Second, there might have been an underreporting of neurological findings, as neuroimaging is not done in all patients, especially those with mild disease. Third, as this is still an emerging disease and there are only a few hundred cases in the literature on neurological manifestations in COVID-19 (refer Supplementary Table S1), we believe further studies on this topic are still required to prove the validity of our results, as bias can be a contributing factor. Fourth, pertaining to our study design, our reported frequencies may not be generalizable to the population and hence there may be a limited external validity as well as a presence of publication bias due to more publications specific to this topic. It would thus be challenging to imply how frequent the imaging findings were among the patients with COVID-19. Hence, these findings should be interpreted with caution. Finally, some relevant studies might have been missed or some studies with unremarkable findings might have gone unpublished.

As the number of cases of COVID-19 is increasing rapidly, there is a rapid emergence of studies showing neuroimaging findings. We now know that neurological manifestations and neuroimaging findings are not uncommon with the disease. We found that ischemic stroke was associated with fatality in COVID-19 infection. Our review also indicates that older patients are at higher risk of stroke. Based on these findings, we recommend the usage of early neuroimaging studies, especially MRI, to be performed in severe COVID-19 patients with neurological complications or unexplained neurological findings, in order to provide early and aggressive intervention if needed. Further studies and data are required to provide a substantial basis and formulation for guidelines on the early implementation of MRI in COVID-19 patients.

## Figures and Tables

**Table 1:** General characteristics of COVID-19 (*n* = 171) patients with neurological manifestation.

Characteristics	N (%)
**Mean Age, SD, IQR**	53.2, 15.5, 40–64
Gender *	159
Male, *n* (%)	106 (66.7)
Female, *n* (%)	53 (33.7)
**Neurological manifestation**	171
Ischemic stroke, *n* (%)	62 (36.2)
Hemorrhagic stroke, *n* (%)	41 (24)
Encephalitis, *n* (%)	24 (14)
CNS inflammatory, *n* (%)	44 (25.7)
Encephalopathy, *n* (%)	11 (6.4)
GBS and its variant, *n* (%)	3 (2)
Olfactory bulb enhancement, *n* (%)	3 (2)
Other cranial nerve enhancement, *n* (%)	6 (3.5)
Others **, *n* (%)	6 (3.5)
Severity of COVID-19 ***	171
Severe	96 (56.2)
Non-Severe	75 (43.8)
**Outcomes**	171
Fatal	49 (28.7)
Non-fatal	122 (71.3)

Abbreviations: SD, Standard Deviation; IQR, Interquartile Range. * Gender data on 12 cases not available. ** Other includes pachymeningeal enhancement and nonspecific findings such as FLAIR hyperintensities in deep white matter. *** Severity based on Infectious Disease Society of America/American Thoracic Society. IDSA/ATS criteria.

**Table 2:** Severity and outcomes of COVID-19 based on neurological pathophysiology confirmed through MRI findings for patients (*n* = 171).

Pathophysiology	N	Severe *n* (%)	Non-Severe *n* (%)	*p*-Value	Fatal *n* (%)	Non-Fatal *n* (%)	*p*-Value
Ischemic stroke Non-ischemic stroke	62 109	35(36) 61(64)	27(36) 48(64)	0.951	25(51) 24(49)	37(30) 85(70)	0.011 *
Hemorrhagic stroke Non-hemorrhagic stroke	41 130	30(31) 66(69)	11(15) 64(85)	0.012 *	17(35) 32(65)	24(20) 98(80)	0.037 *
Encephalitis Non-encephalitis	24 147	17(18) 79(82)	7(9) 68(91)	0.118	3(6) 46(94)	21(17) 101(83)	0.059
CNS inflammatory Non-inflammatory	44 127	24(25) 72(75)	20(27) 55(73)	0.805	11(22) 38(78)	33(27) 89(73)	0.534
Encephalopathy Non-encephalopathy	11 160	11(11) 85(89)	0(0) 75(100)	0.002 *	6(12) 43(88)	5(4) 117(96)	0.049 *
GBS and its variant Non-GBS	3 168	1(1) 95(99)	2(3) 73(97)	0.422	0(0) 49(100)	3(2) 119(98)	0.268
Olfactory bulb enhancement No olfactory bulb enhancement	3 168	0(0) 96(100)	3(4) 72(96)	0.048 *	0(0) 49(100)	3(2) 119(98)	0.268
Other cranial nerve enhancement Non-cranial nerve enhancement	6 165	0(0) 96(100)	6(8) 69(92)	0.005 *	0(0) 49(100)	6(5) 116(95)	0.114
Others (Yes) Others (No)	6 165	4(4) 92(96)	2(3) 73(97)	0.597	3(6) 46(94)	3(2) 119(98)	0.239

* *p*-value significant (bold) at <0.05.

**Table 3:** Age distributions of all COVID-19 patients based on neurological diagnoses through MRI findings.

Characteristics	*n* = 170	Age in Years		*p*-Value
		Mean	SD	
**Severity**				
Yes	96	54.5	14.7	0.305
No	74	51.5	16.5	
**Fatality**				
Yes	49	55.2	15.8	0.331
No	121	52.4	15.4	
**Ischemic stroke**				
Yes	62	57.5	15.4	**0.005** *
No	108	50.7	15.1	
**Hemorrhagic stroke**				
Yes	41	51.2	13.1	0.226
No	129	53.8	16.2	
**Encephalitis**				
Yes	23	48.5	13.8	0.108
No	147	53.9	15.7	
**Encephalopathy**				
Yes	11	50.3	11.8	0.406
No	159	53.4	15.8	
**CNS inflammation**				
Yes	44	53.3	15.8	0.859
No	126	53.2	15.5	
**Olfactory bulb enhancement**				
Yes	3	29.0	5.3	**0.011** *
No	167	53.6	15.3	
**Cranial nerve enhancement**				
Yes	6	49.2	19.7	0.657
No	164	53.3	15.4	
**GBS and its variant**				
Yes	3	51.7	13.7	0.836
No	167	53.2	15.6	
**Others**				
Yes	6	62.3	10.3	0.147
No	164	52.9	15.6	

* p-value significant at <0.05. Others include pachymeningeal enhancement and nonspecific findings such as FLAIR hyperintensities in deep white matter.

**Table 4:** Severity and outcome status of COVID-19 by type of neurological pathophysiology (*n* = 171).

Pathophysiology	N	Severe *n* (%)	Non-Severe *n* (%)	*p*-Value	Fatal *n* (%)	Non-Fatal *n* (%)	*p*-Value
Vascular Non-vascular	90 81	53(59) 43(53)	37(41) 38(47)	0.445	34(38) 15(19)	56(62) 66(81)	**0.005** *
Inflammatory Non-inflammatory	57 114	36(63) 60(53)	21(37) 54(47)	0.191	17(30) 32(28)	40(70) 82(72)	0.811

* *p*-value significant (bold) at <0.05.

**Table 5:** Studies with grouped data with detailed MRI findings and associated neurological manifestations with severity and outcomes for COVID-19.

Author/Country All Studies from Year/Year Published 2020	No. of Patients with Neuroimaging Findings	Neurological Manifestation CNS = 1, PNS = 2	MRI Findings	Severity of COVID-19 * (Non-Severe = 1, Severe = 2)	Outcome (Non-Fatal = 1, Fatal = 2)
Freeman C.W et al. [17]/USA	59	1 = 59 2 = 0	MS = 3 Small vessel ischemic = 23 Acute infarction = 6 Subacute infarction = 4 Chronic infarction = 4 Basal ganglia hypoxia = 1 Microhemorrhage = 4 CRDL = 6	NA	NA
Chougar L. et al. [9]/France	73	1 = 68 2 = 2	Acute infarction = 17 Venous infarction = 1 Microhemorrhage = 8 Perfusion abnormalities = 22 Corpus callosum cytotoxic = 3 Hypoxic Ischemic lesion = 3 Non-specific white matter and BG changes = 8 PRES = 2 Metabolic abnormalities = 3 Neuritis = 2 Meningeal enhancement = 3 Corticospinal tract flair hyperintensity = 1	NA	NA
Conklin J. et al. [18] (c)/USA	11	1 = 11 2 = 0	Microbleed = Punctate and linear SWI lesions in the subcortical and deep white matter = 11	NA	1 = 15 2 = 1
Hernández- Fernández F. et al. [19] (c)/Spain	23	1 = 23 2 = 0	cerebral ischemia = 17 Intracerebral hemorrhage = 5 PRES = 1	1 = 6 2 = 17	1 = 15 2 = 8
Helms J. et al. [3] (c)/France	13	1 = 13 2 = 0	Leptomeningeal enhancement = 8/13 Perfusion abnormalities = 11/11 Ischemic stroke = 3/13	NA	NA
Giorgianni A. et al. [20]/Italy	26	1 = 20 2 = 0	Ischemic = 4 Cerebral hemorrhage = 5 Encephalitis = 1 Non-acute changes = 16	NA	NA
Kremer S. et al. [8] (c)/France	37	1 = 37 2 = 0	Non-hemorrhagic = 17 Hemorrhagic = 20	NA	1 = 32 2 = 5
Feugeas MCH. et al. [21] (c)/France	25	1 = 22 2 = 3	PRES =8 Brain infarcts = 7 Microbleeds = 4 Hemorrhagic transformation of brain infarct = 1 Subacute subdural hematoma = 1 Old deep-brain hematoma = 1 Moderate or marked enlargement of optic nerve sheaths = 3	NA	1 fatal rest NA
Jain R. et al. [22] (c)/USA	38	1 = 38 2 = 0	Large infarct = 17 Lacunar = 9 hemorrhagic stroke = 9 Encephalitis = 1 Hypoxic ischemia = 2	NA	1 = 22 2 = 16
Lin E. et al. [10] (c)/USA	58	1 = 52 2 = 6	Cerebral infarctions = 31 Parenchymal hematoma = 10 Cranial nerve = 6 Critical illness–associated Microhemorrhage = 3 PRES = 3 Non-traumatic SDH = 3 SAH = 2	NA	NA
Delorme C. et al. [23] (c)/France	4	1 = 4	Encephalopathy = 4 FDG-PET/CT pattern of abnormalities, namely frontal hypometabolism and cerebellar hypermetabolism.	NA	1 = 4
Scullen T. et al. [24] (c)/USA	27	1 = 27 2 = 0	COVID-19-associated encephalopathy = 20 COVID-19–associated acute necrotizing encephalopathy = 2 COVID-19–associated vasculopathy = 5	NA	NA
Radmanesh A. et al. [27] (c)/USA	205	1 = 205 2 = 0	Nonspecific white matter microangiopathy = 134 Chronic infarct = 47 Acute/subacute infarct = 13 ICH=11	NA	NA
Mahammedi A. et al. [5] (c)/Italy	51	1 = 48 2 = 3	Acute ischemic infarct = 34 Intracerebral hemorrhage = 6 C.N enhancement = 1 Cauda Equina = 2 Acute encephalopathy = 1 PRES = 1 Nonspecific encephalopathy = 2 MS exacerbation = 2 Cerebral venous thrombosis = 2	NA	NA
Kandemirli S. et al. [26] (c)/Turkey	27	1 = 12 2 = 0	CVT = 1 Acute ischemic infarction = 1 Cortical FLAIR MRI signal intensity abnormality =10	NA	NA
Radmanesh A. et al. [25] (c)/USA	27	1 = 27 2 = 0	Diffuse Leukoencephalopathy = 4 Microbleed = 1 Combination of both = 5 Acute/subacute infarct = 11 ICH = 4 Presumed AHNE = 1	11/27	6/11

* Severity based on Infectious Disease Society of America/American Thoracic Society. IDSA/ATS criteria.

## Supplementary Materials

The following are available online at https://www.mdpi.com/2076-3425/10/12/1017/s1, Table S1: Complete reference list of included studies in the analysis.

## Abbreviations

ADEM	Acute Disseminated Encephalomyelitis
AHNE	Acute Hemorrhagic Necrotizing Encephalopathy
CNS	Central Nervous System
CT	Computed Tomography
COVID-19	Coronavirus infectious disease -2019
CLOCC	Cytotoxic Lesion of Corpus Callosum
GBS	Guillain-Barré Syndrome
IDSA/ATS	Infectious Disease Society of America/American Thoracic Society
MRI	Magnetic Resonance Imaging
MERS	Mild encephalitis/encephalopathy with reversible splenial lesion
MFS	Miller-Fisher syndrome
MS	Multiple Sclerosis
nCov	Novel Coronavirus
ON	Optic Neuritis
PNS	Peripheral Nervous System
PET	Positron emission tomography
PRES	Posterior Reversible Encephalopathy Syndrome
PRISMA	Preferred Reporting Items for Systematic reviews and Meta-Analyses
RT-PCR	Reverse Transcription Polymerase Chain Reaction
SARS-CoV-2	Severe Acute Respiratory Distress Syndrome coronavirus 2

## References

[1] Gardner L. https://coronavirus.jhu.edu/map.html.

[2] Wang Y., Li X., Ren L., Zhao J., Hu Y., Zhang L., Fan G., Xu J., Gu X., Cheng Z. (2020). Clinical features of patients infected with 2019 novel coronavirus in Wuhan, China. Lancet.

[3] Helms J., Kremer S., Merdji H., Clere-Jehl R., Schenck M., Kummerlen C., Collange O., Boulay C., Fafi-Kremer S., Ohana M. (2020). Neurologic Features in Severe SARS-CoV-2 Infection. N. Engl. J. Med..

[4] Berger J.R. (2020). COVID-19 and the nervous system. J. Neurovirol..

[5] Saba L., Vagal A., Leali M., Rossi A., Gaskill M., Sengupta S., Zhang B., Carriero A., Bachir S., Crivelli P. (2020). Imaging in Neurological Disease of Hospitalized COVID-19 Patients: An Italian Multicenter Retrospective Observational Study. Radiology.

[6] Mao L., Jin H., Wang M., Hu Y., Chen S., He Q., Chang J., Hong C., Zhou Y., Wang D. (2020). Neurologic Manifestations of Hospitalized Patients With Coronavirus Disease 2019 in Wuhan, China. JAMA Neurol..

[7] Munhoz R.P., Pedroso J.L., Nascimento F.A., Almeida S.M.D., Barsottini O.G.P., Cardoso F.E.C., Teive H.A.G. (2020). Neurological complications in patients with SARS-CoV-2 infection: A systematic review. Arq. De Neuro-Psiquiatr..

[8] Kremer S., Lersy F., De Sèze J., Ferré J.-C., Maamar A., Carsin-Nicol B., Collange O., Bonneville F., Adam G., Martin-Blondel G. (2020). Brain MRI Findings in Severe COVID-19: A Retrospective Observational Study. Radiology.

[9] Chougar L., Shor N., Weiss N., Galanaud D., Leclercq D., Mathon B., Belkacem S., Stroër S., Burrel S., Boutolleau D. (2020). Retrospective observational study of brain magnetic resonance imaging findings in patients with acute SARS-CoV-2 infection and neurological manifestations. Radiology.

[10] Lin E., Lantos J., Strauss S., Phillips C., Campion T.R., Navi B.B., Parikh N.S., Merkler A.E., Mir S., Zhang C. (2020). Brain Imaging of Patients with COVID-19: Findings at an Academic Institution during the Height of the Outbreak in New York City. Am. J. Neuroradiol..

[11] Baig A.M., Khaleeq A., Ali U., Syeda H. (2020). Evidence of the COVID-19 Virus Targeting the CNS: Tissue Distribution, Host–Virus Interaction, and Proposed Neurotropic Mechanisms. ACS Chem. Neurosci..

[12] Chu H., Chan J.F., Yuen T.T., Shuai H., Yuan S., Wang Y., Hu B., Yip C.C., Tsang J.O., Huang X. (2020). Comparative tropism, replication kinetics, and cell damage profiling of SARS-CoV-2 and SARS-CoV with implications for clinical manifestations, transmissibility, and laboratory studies of COVID-19: An observational study. Lancet Microbe.

[13] Mehta P., McAuley D.F., Brown M., Sanchez E., Tattersall R.S., Manson J.J. (2020). COVID-19: Consider cytokine storm syndromes and immunosuppression. Lancet.

[14] Metlay J.P., Waterer G.W., Long A.C., Anzueto A., Brozek J., Crothers K., Cooley L.A., Dean N.C., Fine M.J., Flanders S.A. (2019). Faculty Opinions recommendation of Diagnosis and Treatment of Adults with Community-acquired Pneumonia. An Official Clinical Practice Guideline of the American Thoracic Society and Infectious Diseases Society of America. Fac. Opin. Post-Publ. Peer Rev. Biomed. Lit..

[15] Moher D., Liberati A., Tetzlaff J., Altman D.G., The PRISMA Group (2009). Preferred reporting items for systematic reviews and meta-analyses: The PRISMA statement. PLoS Med..

[16] Joanna Briggs I. (2019). The Joanna Briggs Institute Critical Appraisal Tools for Use in JBI Systematic Review: Checklists for Case Reports.

[17] Freeman C.W., Masur J., Hassankhani A., Wolf R.L., Levine J.M., Mohan S. (2020). COVID-19-Related Disseminated Leukoencephalopathy (CRDL): A Retrospective Study of Findings on Brain MRI. Am. J. Roentgenol..

[18] Conklin J., Frosch M.P., Mukerji S., Rapalino O., Maher M., Schaefer P.W., Lev M.H., Gonzalez R.G., Das S., Champion S.N. Cerebral Microvascular Injury in Severe COVID-19. medRxiv.

[19] Hernández-Fernández F., Valencia H.S., Barbella-Aponte R.A., Collado-Jiménez R., Ayo-Martín Ó., Barrena C., Molina-Nuevo J.D., García-García J., Lozano-Setién E., Alcahut-Rodriguez C. (2020). Cerebrovascular disease in patients with COVID-19: Neuroimaging, histological and clinical description. Brain.

[20] Giorgianni A., Vinacci G., Agosti E., Mercuri A., Baruzzi F. (2020). Neuroradiological features in COVID-19 patients: First evidence in a complex scenario. J. Neuroradiol..

[21] Feugeas M.-C.H., Gaudemer A., Lescure X., Dossier A., Sonneville R., Ehmer C., Choquet C., Raynaud-Simon A., Borie R., Amarenco P. (2020). Covid-19 and dysregulated cerebral perfusion: Observations with multimodal MRI. medRxiv.

[22] Jain R., Young M., Dogra S., Kennedy H., Nguyen V., Jones S., Bilaloglu S., Hochman K., Raz E., Galetta S. (2020). COVID-19 related neuroimaging findings: A signal of thromboembolic complications and a strong prognostic marker of poor patient outcome. J. Neurol. Sci..

[23] Delorme C., Paccoud O., Kas A., Hesters A., Bombois S., Shambrook P., Boullet A., Doukhi D., Le Guennec L., Godefroy N. (2020). COVID-19-related encephalopathy: A case series with brain FDG-positron-emission tomography/computed tomography findings. Eur. J. Neurol..

[24] Scullen T., Keen J., Mathkour M., Dumont A.S., Kahn L. (2020). COVID-19 Associated Encephalopathies and Cerebrovascular Disease: The New Orleans Experience. World Neurosurgery. https://www.researchgate.net/profile/Mansour_Mathkour/publication/341721713_COVID-19_Associated_Encephalopathies_and_Cerebrovascular_Disease_the_New_Orleans_Experience/links/5ed2a8f392851c9c5e678d34/COVID-19-Associated-Encephalopathies-and-Cerebrovascular-Disease-the-New-Orleans-Experience.pdf.

[25] Radmanesh A., Derman A., Lui Y.W., Raz E., Loh J.P., Hagiwara M., Borja M.J., Zan E., Fatterpekar G. (2020). COVID-19–associated Diffuse Leukoencephalopathy and Microhemorrhages. Radiol..

[26] Kandemirli S.G., Dogan L., Sarıkaya Z.T., Kara S., Akinci C., Kaya D., Kaya Y., Yildirim D., Tuzuner F., Yildirim M.S. (2020). Brain MRI Findings in Patients in the Intensive Care Unit with COVID-19 Infection. Radiology.

[27] Radmanesh A., Raz E., Zan E., Derman A., Kaminetzky M. (2020). Brain Imaging Use and Findings in COVID-19: A Single Academic Center Experience in the Epicenter of Disease in the United States. Am. J. Neuroradiol..

[28] Romero-Sánchez C.M., Díaz-Maroto I., Fernández-Díaz E., Sánchez-Larsen Á., Layos-Romero A., García-García J., González E., Redondo-Peñas I., Perona-Moratalla A.B., Del Valle-Pérez J.A. (2020). Neurologic manifestations in hospitalized patients with COVID-19: The ALBACOVID registry. Neurology.

[29] Garg R.K., Paliwal V.K., Gupta A. (2020). Encephalopathy in patients with COVID-19: A review. J. Med Virol..

[30] Tan Y.-K., Goh C., Leow A.S.T., Tambyah P.A., Ang A., Yap E.-S., Tu T.-M., Sharma V.K., Yeo L.L., Chan B.P.L. (2020). COVID-19 and ischemic stroke: A systematic review and meta-summary of the literature. J. Thromb. Thrombolysis.

[31] Pérez C.A. (2020). Looking ahead: The risk of neurologic complications due to COVID-19. Neurol. Clin. Pract..

[32] Dogra S., Jain R., Cao M., Bilaloglu S., Zagzag D., Hochman S., Lewis A., Melmed K., Hochman K., Horwitz L.I. (2020). Hemorrhagic stroke and anticoagulation in COVID-19. J. Stroke Cerebrovascular Dis..

[33] Pan S., Chen W.C., Baal J.D., Sugrue L.P. (2020). Neuroradiological Features of Mild and Severe SARS-CoV-2 Infection. Acad. Radiol..

[34] Benussi A., Pilotto A., Premi E., Libri I., Giunta M., Agosti C., Alberici A., Baldelli E., Benini M., Bonacina S. (2020). Clinical characteristics and outcomes of inpatients with neurologic disease and COVID-19 in Brescia, Lombardy, Italy. Neurology.

[35] García L.F. (2020). Immune Response, Inflammation, and the Clinical Spectrum of COVID-19. Front. Immunol..

[36] Morris S.B., Schwartz N.G., Patel P., Abbo L., Beauchamps L., Balan S., Lee E.H., Paneth-Pollak R., Geevarughese A., Lash M.K. (2020). Case Series of Multisystem Inflammatory Syndrome in Adults Associated with SARS-CoV-2 Infection—United Kingdom and United States, March–August 2020. Morb. Mortal. Wkly. Rep..

[37] Lechien J.R., Chiesa-Estomba C.M., De Siati D.R., Horoi M., Le Bon S.D., Rodriguez A., Dequanter D., Blecic S., El Afia F., Distinguin L. (2020). Olfactory and gustatory dysfunctions as a clinical presentation of mild-to-moderate forms of the coronavirus disease (COVID-19): A multicenter European study. Eur. Arch. Otorhinolaryngol..

[38] Aragao M., Gouveia M.D.C.L., Filho O.C., Fonseca T., Valenca M. (2020). Anosmia in COVID-19 Associated with Injury to the Olfactory Bulbs Evident on MRI. Am. J. Neuroradiol..

[39] Feign V., Lawes C.M.M., Bennett D.A., Anderson C.S. (2003). Stroke epidemiology: A review of population-based studies of incidence, prevalence, and case-fatality in the late 20th century. Lancet Neurol..

[40] Lee Y., Min P., Lee S., Kim S.-W. (2020). Prevalence and Duration of Acute Loss of Smell or Taste in COVID-19 Patients. J. Korean Med. Sci..

