# Supplementary material for: Relevance and Clinical Significance of Magnetic Resonance Imaging of Neurological Manifestations in COVID-19: A Systematic Review of Case Reports and Case Series
Source: Brain Sci. 2020 Dec 21;10(12):1017. doi: 10.3390/brainsci10121017 (PMC7766893; doi:10.3390/brainsci10121017)
Supplement: Supplementary file 1 [file brainsci-10-01017-s001.pdf]

**Supplementary Table S1: Complete reference list of included studies in the analysis.**

| | |
|---|---|
| 1 | Benger, M., Williams, O., Siddiqui, J., & Sztriha, L. (2020). Intracerebral haemorrhage and COVID-19: Clinical characteristics from a case series. Brain, behavior, and immunity, 88, 940–944. https://doi.org/10.1016/j.bbi.2020.06.005 |
| 2 | Agarwal A, Pinho M, Raj K, Frank FY, Bathla G, Achilleos M, ONeill T, Still M, Maldjian J. Neurological emergencies associated with COVID-19: stroke and beyond. Emergency radiology. 2020 Dec;27(6):747-54. |
| 3 | Cavalcanti DD, Raz E, Shapiro M, Dehkharghani S, Yaghi S, Lillemoe K, Nossek E, Torres J, Jain R, Riina HA, Radmanesh A. Cerebral venous thrombosis associated with COVID-19. American Journal of Neuroradiology. 2020 Aug 1;41(8):1370-6. |
| 4 | Hanafi R, Roger PA, Perin B, Kuchcinski G, Deleval N, Dallery F, Michel D, Hacein-Bey L, Pruvo JP, Outteryck O, Constans JM. COVID-19 neurologic complication with CNS vasculitis-like pattern. American Journal of Neuroradiology. 2020 Aug 1;41(8):1384-7. |
| 5 | Hayashi M, Sahashi Y, Baba Y, Okura H, Shimohata T. COVID-19-associated mild encephalitis/encephalopathy with a reversible splenial lesion. Journal of the Neurological Sciences. 2020 May 27. |
| 6 | Sharifi-Razavi A, Karimi N, Rouhani N. COVID-19 and intracerebral haemorrhage: causative or coincidental?. New microbes and new infections. 2020 May;35. |
| 7 | Moriguchi T, Harii N, Goto J, Harada D, Sugawara H, Takamino J, Ueno M, Sakata H, Kondo K, Myose N, Nakao A. A first case of meningitis/encephalitis associated with SARS-Coronavirus-2. International Journal of Infectious Diseases. 2020 Apr 3. |
| 8 | Poyiadji N, Shahin G, Noujaim D, Stone M, Patel S, Griffith B. COVID-19–associated acute hemorrhagic necrotizing encephalopathy: CT and MRI features. Radiology. 2020 Mar 31:201187. |
| 9 | Radmanesh A, Derman A, Lui YW, Raz E, Loh JP, Hagiwara M, Borja MJ, Zan E, Fatterpekar GM. COVID-19–associated diffuse leukoencephalopathy and microhemorrhages. Radiology. 2020 Oct;297(1):E223. |
| 10 | McCuddy M, Kelkar P, Zhao Y, Wicklund D. Acute Demyelinating Encephalomyelitis (ADEM) in COVID-19 infection: A Case Series. medRxiv. 2020 Jan 1. |
| 11 | Franceschi AM, Arora R, Wilson R, Giliberto L, Libman RB, Castillo M. Neurovascular Complications in COVID-19 Infection: Case Series. American Journal of Neuroradiology. 2020 Jun 11. |
| 12 | Djellaoui A, Seddik L, De Langavant LC, Cattan S, Bachoud-Lévi AC, Hosseini H. Posterior reversible encephalopathy syndrome associated with SARS-CoV-2 infection. Journal of Neurology, Neurosurgery & Psychiatry. 2020 Jul 26. |
| 13 | Lang M, Buch K, Li MD, Mehan WA, Lang AL, Leslie-Mazwi TM, Rincon SP. Leukoencephalopathy associated with severe COVID-19 infection: sequela of hypoxemia?. American Journal of Neuroradiology. 2020 Sep 1;41(9):1641-5. |
| 14 | Munz M, Wessendorf S, Koretsis G, Tewald F, Baegi R, Krämer S, Geissler M, Reinhard M. Acute transverse myelitis after COVID-19 pneumonia. Journal of Neurology. 2020 May 26:1. |
| 15 | Brun G, Hak JF, Coze S, Kaphan E, Carvelli J, Girard N, Stellmann JP. COVID-19—White matter and globus pallidum lesions: Demyelination or small-vessel vasculitis?. Neurology-Neuroimmunology Neuroinflammation. 2020 Jul 1;7(4). |
| 16 | Dixon L, Varley J, Gontsarova A, Mallon D, Tona F, Muir D, Luqmani A, Jenkins IH, Nicholas R, Jones B, Everitt A. COVID-19-related acute necrotizing encephalopathy with brain stem involvement in a patient with aplastic anemia. Neurology-Neuroimmunology Neuroinflammation. 2020 Sep 3;7(5). |
| 17 | Vaschetto R, Cena T, Sainaghi PP, Meneghetti G, Bazzano S, Vecchio D, Pirisi M, Brustia D, Barini M, Cammarota G, Castello L. Cerebral nervous system vasculitis in a Covid-19 patient with pneumonia. Journal of Clinical Neuroscience. 2020 Sep 1;79:71-3. |
| 18 | Zayet S, Klopfenstein T, Kovẚcs R, Stancescu S, Hagenkötter B. Acute cerebral stroke with multiple infarctions and COVID-19, France, 2020. Emerging infectious diseases. 2020 Sep;26(9):2258-60. |
| 19 | Fitsiori A, Pugin D, Thieffry C, Lalive P, Vargas MI. COVID-19 is Associated with an Unusual Pattern of Brain Microbleeds in Critically Ill Patients. Journal of Neuroimaging. 2020 Sep;30(5):593-7. |
| 20 | Fayed I, Pivazyan G, Conte AG, Chang J, Mai JC. Intracranial hemorrhage in critically ill patients hospitalized for COVID-19. Journal of Clinical Neuroscience. 2020 Nov 1;81:192-5. |
| 21 | Forestier G, de Beaurepaire I, Bornet G, Boulouis G. Cytotoxic lesion of the corpus callosum as presenting neuroradiological manifestation of COVID-2019 infection. Journal of Neurology. 2020 Aug 18:1-3. |
| 22 | Maideniuc C, Memon AB. Acute necrotizing myelitis and acute motor axonal neuropathy in a COVID-19 patient. Journal of neurology. 2020 Aug 9:1-3. |
| 23 | Sugiyama Y, Tsuchiya T, Tanaka R, Ouchi A, Motoyama A, Takamoto T, Hara N, Yanagawa Y. Cerebral venous thrombosis in COVID-19-associated coagulopathy: A case report. Journal of Clinical Neuroscience. 2020 Sep 1;79:30-2. |
| 24 | Parauda SC, Gao V, Gewirtz AN, Parikh NS, Merkler AE, Lantos J, White H, Leifer D, Navi BB, Segal AZ. Posterior reversible encephalopathy syndrome in patients with COVID-19. Journal of the Neurological Sciences. 2020 Sep 15;416:117019. |
| 25 | Kakadia B, Ahmed J, Siegal T, Jovin TG, Thon JM. Mild encephalopathy with reversible splenium lesion (MERS) in a patient with COVID-19. Journal of Clinical Neuroscience. 2020 Sep 1;79:272-4. |
| 26 | Krett JD, Jewett GA, Elton-Lacasse C, Fonseca K, Hahn C, Au S, Koch MW. Hemorrhagic encephalopathy associated with COVID-19. Journal of neuroimmunology. 2020 Sep 15;346:577326. |
| 27 | Fadakar N, Ghaemmaghami S, Masoompour SM, Yeganeh BS, Akbari A, Hooshmandi S, Ostovan VR. A first case of acute cerebellitis associated with coronavirus disease (COVID-19): a case report and literature review. The Cerebellum. 2020 Dec;19(6):911-4. |
| 28 | Sirous R, Taghvaei R, Hellinger JC, Krauthamer AV, Mirfendereski S. COVID-19-associated encephalopathy with fulminant cerebral vasoconstriction: CT and MRI findings. Radiology Case Reports. 2020 Nov 1;15(11):2208-12. |
| 29 | Degen C, Lenarz T, Willenborg K. Acute profound sensorineural hearing loss after COVID-19 pneumonia. InMayo Clinic Proceedings 2020 Aug 1 (Vol. 95, No. 8, pp. 1801-1803). Elsevier. |
| 30 | Zanin L, Saraceno G, Panciani PP, Renisi G, Signorini L, Migliorati K, Fontanella MM. SARS-CoV-2 can induce brain and spine demyelinating lesions. Acta Neurochirurgica. 2020 May 4:1-4. |
| 31 | Malentacchi M, Gned D, Angelino V, Demichelis S, Perboni A, Veltri A, Bertolotto A, Capobianco M. Concomitant brain arterial and venous thrombosis in a COVID-19 patient. European Journal of Neurology. 2020 Jun 5. |

| | |
|---|---|
| 32 | Kishfy L, Casasola M, Banankhah P, Parvez A, Jan YJ, Shenoy AM, Thomson C, AbdelRazek MA. Posterior reversible encephalopathy syndrome (PRES) as a neurological association in severe Covid-19. Journal of the Neurological Sciences. 2020 Jul 15;414:116943. |
| 33 | Ashrafi F, Zali A, Ommi D, Salari M, Fatemi A, Arab-Ahmadi M, Behnam B, Azhideh A, Vahidi M, Yousefi-Asl M, Advani S. COVID-19-related strokes in adults below 55 years of age: a case series. Neurological Sciences. 2020 Aug;41(8):1985-9. |
| 34 | Morassi M, Bagatto D, Cobelli M, D'Agostini S, Gigli GL, Bnà C, Vogrig A. Stroke in patients with SARS-CoV-2 infection: case series. Journal of Neurology. 2020 May 20:1. |
| 35 | Kaya Y, Kara S, Akinci C, Kocaman AS. Transient cortical blindness in COVID-19 pneumonia; a PRES-like syndrome: Case report. Journal of the Neurological Sciences. 2020 Jun 15;413:116858. |
| 36 | Li J, Long X, Zhu C, Hu S, Lin Z, Li J, Xiong N. A case of COVID-19 pneumonia with cerebral hemorrhage. Thrombosis Research. 2020 May 30. |
| 37 | Kulick-Soper CV, McKee JL, Wolf RL, Mohan S, Stein JM, Masur JH, Lazor JW, Dunlap DG, McGinniss JE, David MZ, England RN. Pearls & Oy-sters: Bilateral globus pallidus lesions in a patient with COVID-19. Neurology. 2020 Sep 8;95(10):454-7. |
| 38 | Dinkin M, Gao V, Kahan J, Bobker S, Simonetto M, Wechsler P, Harpe J, Greer C, Mints G, Salama G, Tsiouris AJ. COVID-19 presenting with ophthalmoparesis from cranial nerve palsy. Neurology. 2020 Apr 28. |
| 39 | Lantos JE, Strauss SB, Lin E. COVID-19–associated miller fisher syndrome: MRI findings. American Journal of Neuroradiology. 2020 Jul 1;41(7):1184-6. |
| 40 | Efe IE, Aydin OU, Alabulut A, Celik O, Aydin K. COVID-19-associated encephalitis mimicking glial tumor: a case report. World Neurosurgery. 2020 May 29. |
| 41 | Novi G, Rossi T, Pedemonte E, Saitta L, Rolla C, Roccatagliata L, Inglese M, Farinini D. Acute disseminated encephalomyelitis after SARS-CoV-2 infection. Neurology-Neuroimmunology Neuroinflammation. 2020 Sep 1;7(5). |
| 42 | Sancho-Saldaña A, Lambea-Gil Á, Liesa JL, Caballo MR, Garay MH, Celada DR, Serrano-Ponz M. Guillain–Barré syndrome associated with leptomeningeal enhancement following SARS-CoV-2 infection. Clinical Medicine. 2020 Jun 9. |
| 43 | Hutchins KL, Jansen JH, Comer AD, Scheer RV, Zahn GS, Capps AE, Weaver LM, Koontz NA. COVID-19–associated bifacial weakness with paresthesia subtype of Guillain-Barré syndrome. American Journal of Neuroradiology. 2020 Sep 1;41(9):1707-11. |
| 44 | Chalil A, Baker CS, Johnston RB, Just C, Debicki DB, Mayich MS, Bosma KJ, Steven DA. Acute Hemorrhagic Encephalitis Related to COVID-19. Neurology: Clinical Practice. 2020 Jul 7. |
| 45 | Morvan AC, Kerambrun H. Fatal necrotizing encephalitis associated with COVID-19: A case report. Neurology: Clinical Practice. 2020 Aug 18. |
| 46 | Elkady A, Rabinstein AA. Acute necrotizing encephalopathy and myocarditis in a young patient with COVID-19. Neurology-Neuroimmunology Neuroinflammation. 2020 Sep 1;7(5). |
| 47 | Abdelhady M, Elsotouhy A, Vattoth S. Acute Flaccid Myelitis in COVID-19. BJR\| case reports. 2020 Sep;6(3):20200098. |
| 48 | Vattoth S, Abdelhady M, Alsoub H, Own A, Elsotouhy A. Critical illness-associated cerebral microbleeds in COVID-19. The Neuroradiology Journal. 2020 Oct;33(5):374-6. |
| 49 | Sotoca J, Rodríguez-Álvarez Y. COVID-19-associated acute necrotizing myelitis. Neurology® Neuroimmunology & Neuroinflammation. 2020 Sep;7(5). |
| 50 | Fu B, Chen Y, Li P. The 2019 novel coronavirus disease with secondary ischemic stroke: two cases report. |
| 51 | Avula A, Nalleballe K, Narula N, Sapozhnikov S, Dandu V, Toom S, Glaser A, Elsayegh D. COVID-19 presenting as stroke. Brain, behavior, and immunity. 2020 Apr 28. |
| 52 | Valderrama EV, Humbert K, Lord A, Frontera J, Yaghi S. Severe acute respiratory syndrome coronavirus 2 infection and ischemic stroke. Stroke. 2020 May 12:STROKEAHA-120. |
| 53 | Aragão MD, Leal MC, Cartaxo Filho OQ, Fonseca TM, Valença MM. Anosmia in COVID-19 associated with injury to the olfactory bulbs evident on MRI. American Journal of Neuroradiology. 2020 Sep 1;41(9):1703-6. |
| 54 | Goh Y, Beh DL, Makmur A, Somani J, Chan AC. Pearls and Oy-sters: Facial nerve palsy as a neurological manifestation of Covid-19 infection. Neurology. 2020 May 20. |
| 55 | Farhadian S, Glick LR, Vogels CB, Thomas J, Chiarella J, Casanovas-Massana A, Zhou J, Odio C, Vijayakumar P, Geng B, Fournier J. Acute encephalopathy with elevated CSF inflammatory markers as the initial presentation of COVID-19. |
| 56 | Abdulkadir TU, ÜNLÜBAŞ Y, ALEMDAR M, AKYÜZ E. Coexistence of COVID-19 and acute ischemic stroke report of four cases. Journal of Clinical Neuroscience. 2020 May 6. |
| 57 | Oxley TJ, Mocco J, Majidi S, Kellner CP, Shoirah H, Singh IP, De Leacy RA, Shigematsu T, Ladner TR, Yaeger KA, Skliut M. Large-vessel stroke as a presenting feature of Covid-19 in the young. New England Journal of Medicine. 2020 May 14;382(20):e60. |
| 58 | Laurendon T, Radulesco T, Mugnier J, Gérault M, Chagnaud C, El Ahmadi AA, Varoquaux A. Bilateral transient olfactory bulb edema during COVID-19–related anosmia. Neurology. 2020 Aug 4;95(5):224-5. |
| 59 | Strauss SB, Lantos JE, Heier LA, Shatzkes DR, Phillips CD. Olfactory bulb signal abnormality in patients with COVID-19 who present with neurologic symptoms. American Journal of Neuroradiology. 2020 Oct 1;41(10):1882-7. |
| 60 | Politi LS, Salsano E, Grimaldi M. Magnetic resonance imaging alteration of the brain in a patient with coronavirus disease 2019 (covid-19) and anosmia. JAMA Neurology. 2020 May 29. |
| 61 | Toscano G, Palmerini F, Ravaglia S, Ruiz L, Invernizzi P, Cuzzoni MG, Franciotta D, Baldanti F, Daturi R, Postorino P, Cavallini A. Guillain–Barré syndrome associated with SARS-CoV-2. New England Journal of Medicine. 2020 Apr 17. |
| 62 | Roy-Gash F, Jean-Michel D, Herve V, Raphael B, Nicolas E. COVID-19-associated acute cerebral venous thrombosis: clinical, CT, MRI and EEG features. Critical Care. 2020 Dec;24(1):1-3. |
| 63 | OLIVEIRA RD, Santos DH, Olivetti BC, Takahashi JT. Bilateral trochlear nerve palsy due to cerebral vasculitis related to COVID-19 infection. Arquivos de Neuro-Psiquiatria. 2020 Jun;78(6):385-6. |
| 64 | Reddy ST, Garg T, Shah C, Nascimento FA, Imran R, Kan P, Bowry R, Gonzales N, Barreto A, Kumar A, Volpi J. Cerebrovascular Disease in Patients with COVID-19: A Review of the Literature and Case Series. Case Reports in Neurology. 2020;12(2):199-209. |
| 65 | Le Guennec L, Devianne J, Jalin L, Cao A, Galanaud D, Navarro V, Boutolleau D, Rohaut B, Weiss N, Demeret S. Orbitofrontal involvement in a neuroCOVID-19 patient. Epilepsia. 2020 Aug;61(8):e90-4. |

| | |
|---|---|
| 66 | Beyrouti R, Adams ME, Benjamin L, Cohen H, Farmer SF, Goh YY, Humphries F, Jäger HR, Losseff NA, Perry RJ, Shah S. Characteristics of ischaemic stroke associated with COVID-19. Journal of Neurology, Neurosurgery & Psychiatry. 2020 Apr 30. |
| 67 | Nicholson P, Alshafai L, Krings T. Neuroimaging Findings in Patients with COVID-19. American Journal of Neuroradiology. 2020 Jun 11. |
| 68 | Fitsiori A, Pugin D, Thieffry C, Lalive d'Epinay P, Vargas Gomez MI. Unusual microbleeds in brain MRI of Covid-19 patients. Journal of Neuroimaging. 2020;30(5):593-7. |
| 69 | Zoghi A, Ramezani M, Roozbeh M, Darazam IA, Sahraian MA. A case of possible atypical demyelinating event of the central nervous system following COVID-19. Multiple sclerosis and related disorders. 2020 Sep 1;44:102324. |
| 70 | Chetrit A, Lechien JR, Ammar A, Chekkoury-Idrissi Y, Distinguin L, Circiu M, Saussez S, Ballester MC, Vasse M, Berradja N, Hans S. Magnetic resonance imaging of COVID-19 anosmic patients reveals abnormalities of the olfactory bulb: preliminary prospective study. The Journal of Infection. 2020 Nov 1. |
| 71 | Coolen T, Lolli V, Sadeghi N, Rovai A, Trotta N, Taccone FS, Creteur J, Henrard S, Goffard JC, Dewitte O, Naeije G. Early postmortem brain MRI findings in COVID-19 non-survivors. medRxiv. 2020 Jan 1. |
| 72 | Anzalone N, Castellano A, Scotti R, Scandroglio AM, Filippi M, Ciceri F, Tresoldi M, Falini A. Multifocal laminar cortical brain lesions: a consistent MRI finding in neuro-COVID-19 patients. Journal of Neurology. 2020 Jun 6:1. |
| 73 | AlKetbi R, AlNuaimi D, AlMulla M, AlTalai N, Samir M, Kumar N, AlBastaki U. Acute myelitis as a neurological complication of Covid-19: a case report and MRI findings. Radiology case reports. 2020 Sep 1;15(9):1591-5. |
| 74 | Sarma D, Bilello LA. A Case Report of Acute Transverse Myelitis Following Novel Coronavirus Infection. Clinical Practice and Cases in Emergency Medicine. 2020 May 12. |
| 75 | Vu D, Ruggiero M, Choi WS, Masri D, Flyer M, Shyknevsky I, Stein EG. Three unsuspected CT diagnoses of COVID-19. Emergency radiology. 2020 Apr 13:1-4. |
| 76 | Bao Y, Lin SY, Cheng ZH, Xia J, Sun YP, Zhao Q, Liu GJ. Clinical Features of COVID-19 in a Young Man with Massive Cerebral Hemorrhage—Case Report. Sn Comprehensive Clinical Medicine. 2020 May 18:1. |
| 77 | Daci R, Kennelly M, Ferris A, Azeem MU, Johnson MD, Hamzei-Sichani F, Jun-O'Connell AH, Natarajan SK. Bilateral basal ganglia hemorrhage in a patient with confirmed COVID-19. American Journal of Neuroradiology. 2020 Oct 1;41(10):1797-9. |
| 78 | Al Mazrouei SS, Saeed GA, Al Helali AA, Ahmed M. COVID-19-associated encephalopathy: Neurological manifestation of COVID-19. Radiology case reports. 2020 Sep 1;15(9):1646-9. |
| 79 | Hemasian H, Ansari B. First case of Covid-19 presented with cerebral venous thrombosis: A rare and dreaded case. Revue Neurologique. 2020 Jun;176(6):521. |
| 80 | Garaci F, Di Giuliano F, Picchi E, Da Ros V, Floris R. Venous cerebral thrombosis in COVID-19 patient. Journal of the Neurological Sciences. 2020 Jul 15;414. |
| 81 | Andrea G, Vinacci G, Edoardo A, Anna M, Fabio B. Neuroradiological features in COVID-19 patients: first evidence in a complex scenario. Journal of Neuroradiology. 2020 May 14. |
| 82 | Franceschi AM, Ahmed O, Giliberto L, Castillo M. Hemorrhagic posterior reversible encephalopathy syndrome as a manifestation of COVID-19 infection. American Journal of Neuroradiology. 2020 Jul 1;41(7):1173-6. |
| 83 | Parsons T, Banks S, Bae C, Gelber J, Alahmadi H, Tichauer M. COVID-19-associated acute disseminated encephalomyelitis (ADEM). Journal of Neurology. 2020 May 30:1. |
| 84 | Goldberg MF, Goldberg MF, Cerejo R, Tayal AH. Cerebrovascular Disease in COVID-19. American Journal of Neuroradiology. 2020 May 14. |
| 85 | Viguier A, Delamarre L, Duplantier J, Olivot JM, Bonneville F. Acute ischemic stroke complicating common carotid artery thrombosis during a severe COVID-19 infection. Journal of neuroradiology. 2020 May 4. |
| 86 | Poillon G, Obadia M, Perrin M, Savatovsky J, Lecler A. Cerebral Venous Thrombosis associated with COVID-19 infection: causality or coincidence?. Journal of neuroradiology. 2020 May 11. |
| 87 | Wong PF, Craik S, Newman P, Makan A, Srinivasan K, Crawford E, Dev D, Moudgil H, Ahmad N. Lessons of the month 1: A case of rhombencephalitis as a rare complication of acute COVID-19 infection. Clinical Medicine. 2020 May 1;20(3):293-4. |
| 88 | Eliezer M, Hautefort C. MRI evaluation of the olfactory clefts in patients with SARS-CoV-2 infection revealed an unexpected mechanism for olfactory function loss. Academic Radiology. 2020 Aug 1;27(8):1191. |
| 89 | Shoskes A, Migdady I, Fernandez A, Ruggieri P, Rae-Grant A. Cerebral Microhemorrhage and Purpuric Rash in COVID-19: The Case for a Secondary Microangiopathy. Journal of Stroke and Cerebrovascular Diseases. 2020 Oct 1;29(10):105111. |
| 90 | Moshayedi P, Ryan TE, Mejia LL, Nour M, Liebeskind DS. Triage of acute ischemic stroke in confirmed COVID-19: large vessel occlusion associated with coronavirus infection. Frontiers in neurology. 2020;11. |
| 91 | Abdi S, Ghorbani A, Fatehi F. The association of SARS-CoV-2 infection and acute disseminated encephalomyelitis without prominent clinical pulmonary symptoms. Journal of the neurological sciences. 2020 Sep 15;416. |
| 92 | Al-olama M, Rashid A, Garozzo D. COVID-19-associated meningoencephalitis complicated with intracranial hemorrhage: a case report. Acta Neurochirurgica. 2020 May 14:1. |
| 93 | Cariddi LP, Damavandi PT, Carimati F, Banfi P, Clemenzi A, Marelli M, Giorgianni A, Vinacci G, Mauri M, Versino M. Reversible encephalopathy syndrome (PRES) in a COVID-19 patient. Journal of neurology. 2020 Nov;267(11):3157-60. |
| 94 | Haddadi K, Ghasemian R, Shafizad M. Basal Ganglia Involvement and Altered Mental Status: A Unique Neurological Manifestation of Coronavirus Disease 2019. Cureus. 2020 Apr;12(4). |
| 95 | Espinosa PS, Rizvi Z, Sharma P, Hindi F, Filatov A. Neurological Complications of Coronavirus Disease (COVID-19): Encephalopathy, MRI Brain and Cerebrospinal Fluid Findings: Case 2. Cureus. 2020 May;12(5). |
| 96 | Li CW, Syue LS, Tsai YS, Li MC, Lo CL, Tsai CS, Chen PL, Ko WC, Lee NY. Anosmia and olfactory tract neuropathy in a case of COVID-19. Journal of Microbiology, Immunology and Infection. 2020 Jun 20. |
| 97 | Muhammad S, Petridisa A, Cornelius JF, Hänggi D. Letter to editor: Severe brain haemorrhage and concomitant COVID-19 Infection: A neurovascular complication of COVID-19. Brain, Behavior, and Immunity. 2020 May 5. |
| 98 | Rogg J, Baker A, Tung G. Posterior reversible encephalopathy syndrome (PRES): Another imaging manifestation of COVID-19. Interdisciplinary Neurosurgery. 2020 Dec 1;22:100808. |

| | |
|---|---|
| 99 | Sachs JR, Gibbs KW, Swor DE, Sweeney AP, Williams DW, Burdette JH, West TG, Geer CP. COVID-19-Associated Leukoencephalopathy. Radiology. 2020 May 14:201753. |
| 100 | Yong MH, Chan YF, Liu J, Sanamandra SK, Kheok SW, Lim KC, Sewa DW. A rare case of acute hemorrhagic leukoencephalitis in a COVID-19 patient. Journal of the neurological sciences. 2020 Sep 15. |
| 101 | Byrnes S, Bisen M, Syed B, Huda S, Siddique Z, Sampat P, Russo R, Oueida Z, Johri G, Dargon I. COVID-19 encephalopathy masquerading as substance withdrawal. Journal of Medical Virology. 2020 May 27. |
| 102 | Nepal P, Batchala PP, Songmen S, Parashar K, Sapire J. An unresponsive COVID-19 patient. Emergency Radiology. 2020 May 28. |
| 103 | Dogan L, Kaya D, Sarikaya T, Zengin R, Dincer A, Akinci IO, Afsar N. Plasmapheresis treatment in COVID-19–related autoimmune meningoencephalitis: Case series. Brain, Behavior, and Immunity. 2020 May 7. |
| 104 | Deliwala S, Abdulhamid S, Abusalih MF, Al-Qasmi MM, Bachuwa G. Encephalopathy as the Sentinel Sign of a Cortical Stroke in a Patient Infected With Coronavirus Disease-19 (COVID-19). Cureus. 2020 May;12(5). |
| 105 | Matos AR, Quintas-Neves M, Oliveira AI, Dias L, Marques S, Carvalho R, Alves JN. Covid-19 associated central nervous system vasculopathy. Canadian Journal of Neurological Sciences. 2020 Jun 2:1-2. |
| 106 | Freeman CW, Masur J, Hassankhani A, Wolf RL, Levine JM, Mohan S. COVID-19-related disseminated leukoencephalopathy (CRDL): a retrospective study of findings on brain MRI. American Journal of Roentgenology. 2020 Sep 9. |
| 107 | Kihira S, Delman BN, Belani P, Stein L, Aggarwal A, Rigney B, Schefflein J, Doshi AH, Pawha PS. Imaging Features of Acute Encephalopathy in Patients with COVID-19: A Case Series. American Journal of Neuroradiology. 2020 Oct 1;41(10):1804-8. |
| 108 | Zambreanu L, Lightbody S, Bhandari M, Hoskote C, Kandil H, Houlihan CF, Lunn MP. A case of limbic encephalitis associated with asymptomatic COVID-19 infection. Journal of Neurology, Neurosurgery & Psychiatry. 2020 Nov 1;91(11):1229-30. |
| 109 | Zuhorn F, Omaimen H, Ruprecht B, Stellbrink C, Rauch M, Rogalewski A, Klingebiel R, Schäbitz WR. Parainfectious encephalitis in COVID-19:"The Claustrum Sign". Journal of Neurology. 2020 Sep 3:1-4. |
| 110 | Grimaldi S, Lagarde S, Harle JR, Boucraut J, Guedj E. Autoimmune encephalitis concomitant with SARS-CoV-2 infection: insight from 18F-FDG PET imaging and neuronal autoantibodies. Journal of Nuclear Medicine. 2020 Jul 24:jnumed-120. |
| 111 | Benameur K, Agarwal A, Auld SC, Butters MP, Webster AS, Ozturk T, Howell JC, Bassit LC, Velasquez A, Schinazi RF, Mullins ME. Early Release-Encephalopathy and Encephalitis Associated with Cerebrospinal Fluid Cytokine Alterations and Coronavirus Disease, Atlanta, Georgia, USA, 2020. |
| 112 | Demirci Otluoglu G, Yener U, Demir MK, Yilmaz B. Encephalomyelitis associated with Covid-19 infection: case report. British journal of neurosurgery. 2020 Jul 6:1-3. |
| 113 | Hoelscher C, Sweid A, Ghosh R, Al Saiegh F, Keppetipola KM, Farrell CJ, Jallo J, Jabbour P, Tjoumakaris S, Gooch MR, Rosenwasser RH. Cerebral deep venous thrombosis and COVID-19: case report. Journal of neurosurgery. 2020 Sep 4;1(aop):1-4. |
| 114 | Delamarre L, Gollion C, Grouteau G, Rousset D, Jimena G, Roustan J, Gaussiat F, Aldigé E, Gaffard C, Duplantier J, Martin C. COVID-19–associated acute necrotising encephalopathy successfully treated with steroids and polyvalent immunoglobulin with unusual IgG targeting the cerebral fibre network. Journal of Neurology, Neurosurgery & Psychiatry. 2020 Sep 1;91(9):1004-6. |
| 115 | Mohamud AY, Griffith B, Rehman M, Miller D, Chebl A, Patel SC, Howell B, Kole M, Marin H. Intraluminal carotid artery thrombus in COVID-19: another danger of cytokine storm?. American Journal of Neuroradiology. 2020 Sep 1;41(9):1677-82. |
| 116 | Toledano-Massiah S, Badat N, Leberre A, Bruel C, Ray A, Gerber S, Zins M, Hodel J. Unusual Brain MRI Pattern in 2 Patients with COVID-19 Acute Respiratory Distress Syndrome. American Journal of Neuroradiology. 2020 Sep 3. |
| 117 | Falcone MM, Rong AJ, Salazar H, Redick DW, Falcone S, Cavuoto KM. Acute abducens nerve palsy in a patient with the novel coronavirus disease (COVID-19). Journal of American Association for Pediatric Ophthalmology and Strabismus. 2020 Jun 24. |
| 118 | Virhammar J, Kumlien E, Fällmar D, Frithiof R, Jackmann S, Sköld MK, Kadir M, Frick J, Lindeberg J, Olivero-Reinius H, Ryttlefors M. Acute necrotizing encephalopathy with SARS-CoV-2 RNA confirmed in cerebrospinal fluid. Neurology. 2020 Sep 8;95(10):445-9. |
| 119 | Vollono C, Rollo E, Romozzi M, Frisullo G, Servidei S, Borghetti A, Calabresi P. Focal status epilepticus as unique clinical feature of COVID-19: A case report. Seizure. 2020 Apr 21. |
| 120 | Afshar H, Yassin Z, Kalantari S, Aloosh O, Lotfi T, Moghaddasi M, Sadeghipour A, Emamikhah M. Evolution and resolution of brain involvement associated with SARS-CoV2 infection: A close Clinical–Paraclinical follow up study of a case. Multiple Sclerosis and Related Disorders. 2020 May 21:102216. |
| 121 | de Sousa GC, de Sousa TC, Sakiyama MA, da Silva JS, de Sousa ED. Vasculitis-related stroke in young as a presenting feature of novel coronavirus disease (COVID19)–Case report. Journal of Clinical Neuroscience. 2020 Sep 1;79:169-71. |
| 122 | Dixon L, Coughlan C, Karunaratne K, Gorgoraptis N, Varley J, Husselbee J, Mallon D, Carroll R, Jones B, Boynton C, Pritchard J. Immunosuppression for intracranial vasculitis associated with SARS-CoV-2: therapeutic implications for COVID-19 cerebrovascular pathology. Journal of Neurology, Neurosurgery & Psychiatry. 2020 Aug 27. |
| 123 | Bolaji P, Kukoyi B, Ahmad N, Wharton C. Extensive cerebral venous sinus thrombosis: a potential complication in a patient with COVID-19 disease. BMJ Case Reports CP. 2020 Aug 1;13(8):e236820. |
| 124 | Rigamonti A, Mantero V, Piamarta F, Spena G, Salmaggi A. Cerebral venous thrombosis associated with coronavirus infection: an underestimated entity?. Neurological Sciences. 2020 Jun 29:1-2. |
| 125 | Hughes C, Nichols T, Pike M, Subbe C, Elghenzai S. Cerebral Venous Sinus Thrombosis as a Presentation of COVID-19. European Journal of Case Reports in Internal Medicine. 2020;7(5). |
| 126 | Beretta S, Da Re F, Francioni V, Remida P, Storti B, Fumagalli L, Piatti ML, Santoro P, Cereda D, Cutellè C, Pirro F. Massive cerebral venous thrombosis related to oligosymptomatic COVID-19 infection: a case report. |
| 127 | Bigliardi G, Ciolli L, Giovannini G, Vandelli L, Dell'Acqua ML, Borzì GM, Picchetto L, Rosafio F, Ricceri R, Meletti S. Middle cerebral artery ischemic stroke and COVID-19: a case report. Journal of neurovirology. 2020 Sep 8:1-3. |
| 128 | Sweid A, Hammoud B, Bekelis K, Missios S, Tjoumakaris SI, Gooch MR, Herial NA, Zarzour H, Romo V, DePrince M, Rosenwasser RH. Cerebral ischemic and hemorrhagic complications of coronavirus disease 2019. International Journal of Stroke. 2020 Oct;15(7):733. |
| 129 | Klein DE, Libman R, Kirsch C, Arora R. Cerebral Venous Thrombosis: Atypical Presentation of COVID-19 in the Young. Journal of Stroke and Cerebrovascular Diseases. 2020 May 23:104989. |

| | |
|---|---|
| 130 | Roy D, Hollingworth M, Kumaria A. A case of malignant cerebral infarction associated with COVID-19 infection. British Journal of Neurosurgery. 2020 Jul 15:1-4. |
| 131 | Guillan M, Villacieros-Alvarez J, Bellido S, Peremarch CP, Suarez-Vega VM, Aragones-Garcia M, Cabrera-Rojo C, Fernandez-Ferro J. Unusual simultaneous cerebral infarcts in multiple arterial territories in a COVID-19 patient. Thrombosis Research. 2020 Jun 9. |
| 132 | Elshereye A, Erdinc B. Multiple Lacunar Cerebral Infarcts as the Initial Presentation of COVID-19. Cureus. 2020 Aug;12(8). |
| 133 | Williams OH, Mohideen S, Sen A, Martinovic O, Hart J, Brex PA, Sztriha LK. Multiple internal border zone infarcts in a patient with COVID-19 and CADASIL. Journal of the Neurological Sciences. 2020 Jun 9. |
| 134 | Mahammedi, A., Saba, L., Vagal, A., Leali, M., Rossi, A., Gaskill, M., ... & Crivelli, P. (2020). Imaging in Neurological Disease of Hospitalized COVID-19 Patients: An Italian Multicenter Retrospective Observational Study. Radiology, 201933-201933. |
